# Supplementary material for: Global determinants of freshwater and marine fish genetic diversity
Source: Nat Commun. 2020 Feb 10;11:692. doi: 10.1038/s41467-020-14409-7 (PMC7010757; doi:10.1038/s41467-020-14409-7)
Supplement: Supplementary file 1 — Supplementary Information [file 41467_2020_14409_MOESM1_ESM.pdf]

Supplementary Information

Global patterns of fish genetic diversity

Manel et al.

.

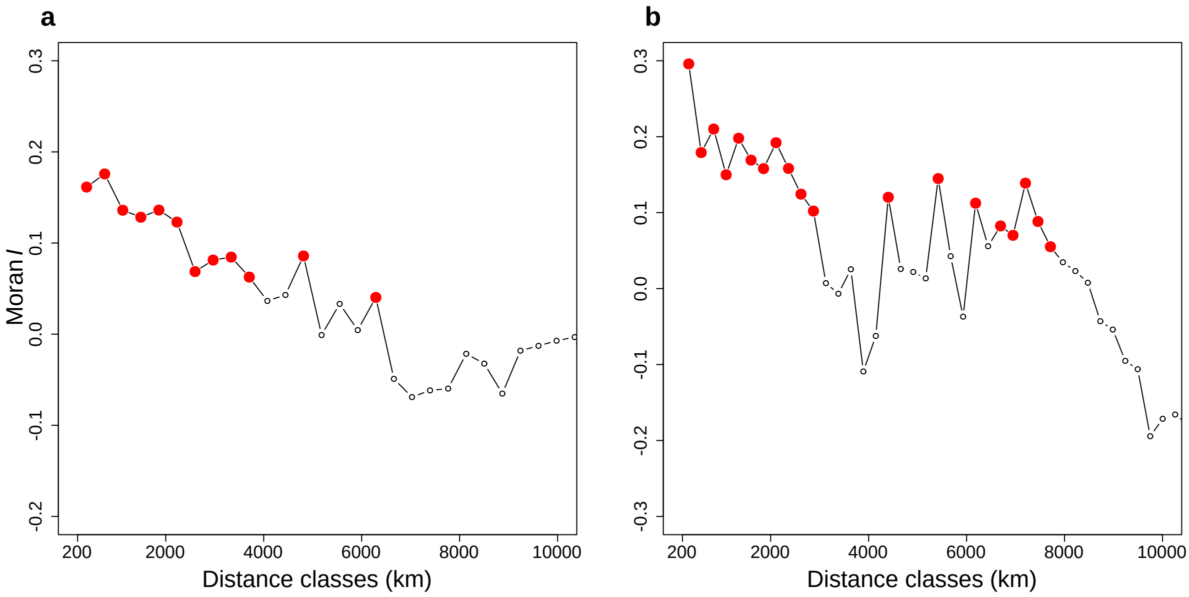

Supplementary Figure 1. Spatial autocorrelogramme based on the *I*-Moran coefficient (R package pgirmess function correlog) of the genetic diversity for marine (a) and freshwater (b) species. Distances classes are in km. Red dots indicates statistically significant values ($p < 0.05$).

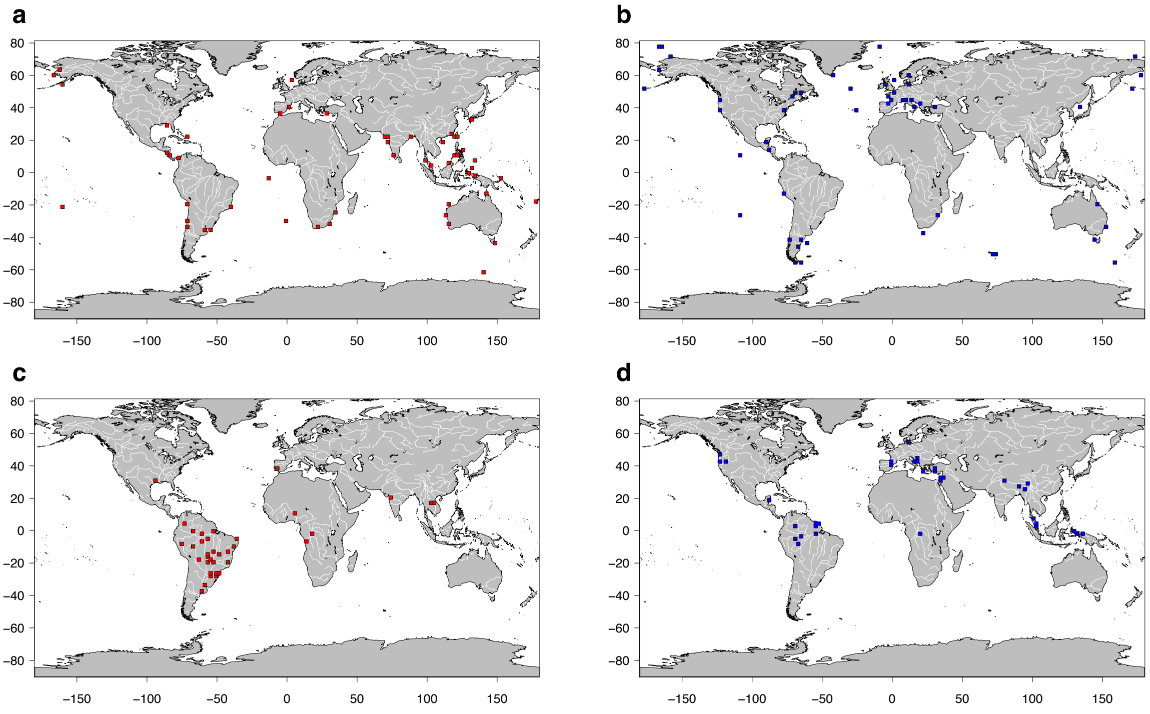

Supplementary Figure 2. Global distribution of higher and lower percentiles of genetic diversity: (a) and (c) cells above the 90th percentile of genetic diversity distribution (= top 10% of richest cells) (in red) and (b) and (d) cells below the 10th percentile (= top worse 10% cells) (in blue) of the distribution of genetic diversity for marine (a-b) and freshwater (c-d) fish species. Drawn with R version 3.2.3.

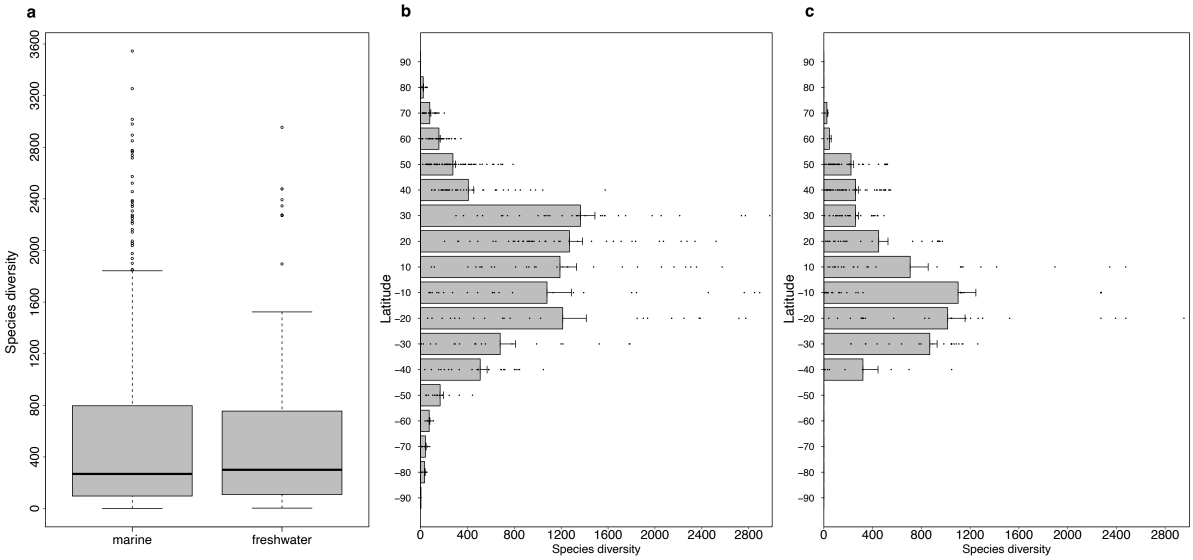

Supplementary Figure 3. Boxplots of species richness (a) were produced with the R command boxplot. Median, upper and lower quartiles and outliers are represented on the boxplot. Latitudinal distribution of species richness for (b) marine and (c) freshwater fish species with error bars indicating confidence interval of species diversity among sites within latitudinal band.

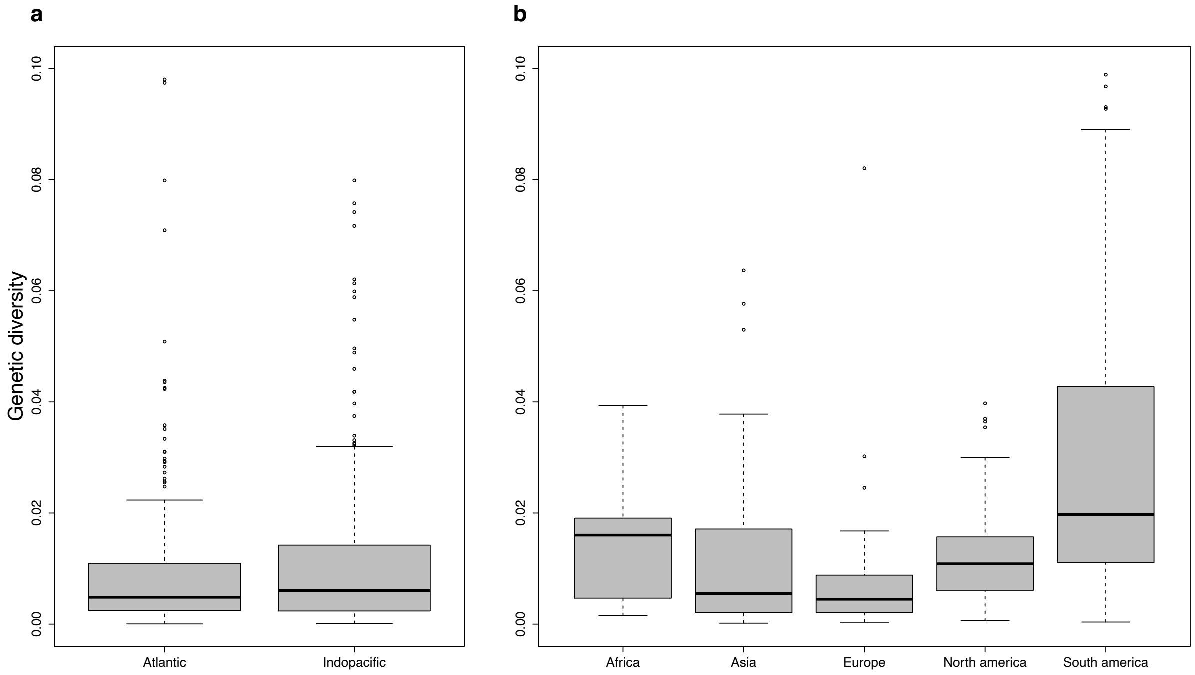

Supplementary Figure 4: Box plots showing the regional effect on the global genetic diversity pattern (a) for marine and (b) freshwater fish species. Median, upper and lower quartiles and outliers are represented on the boxplot.

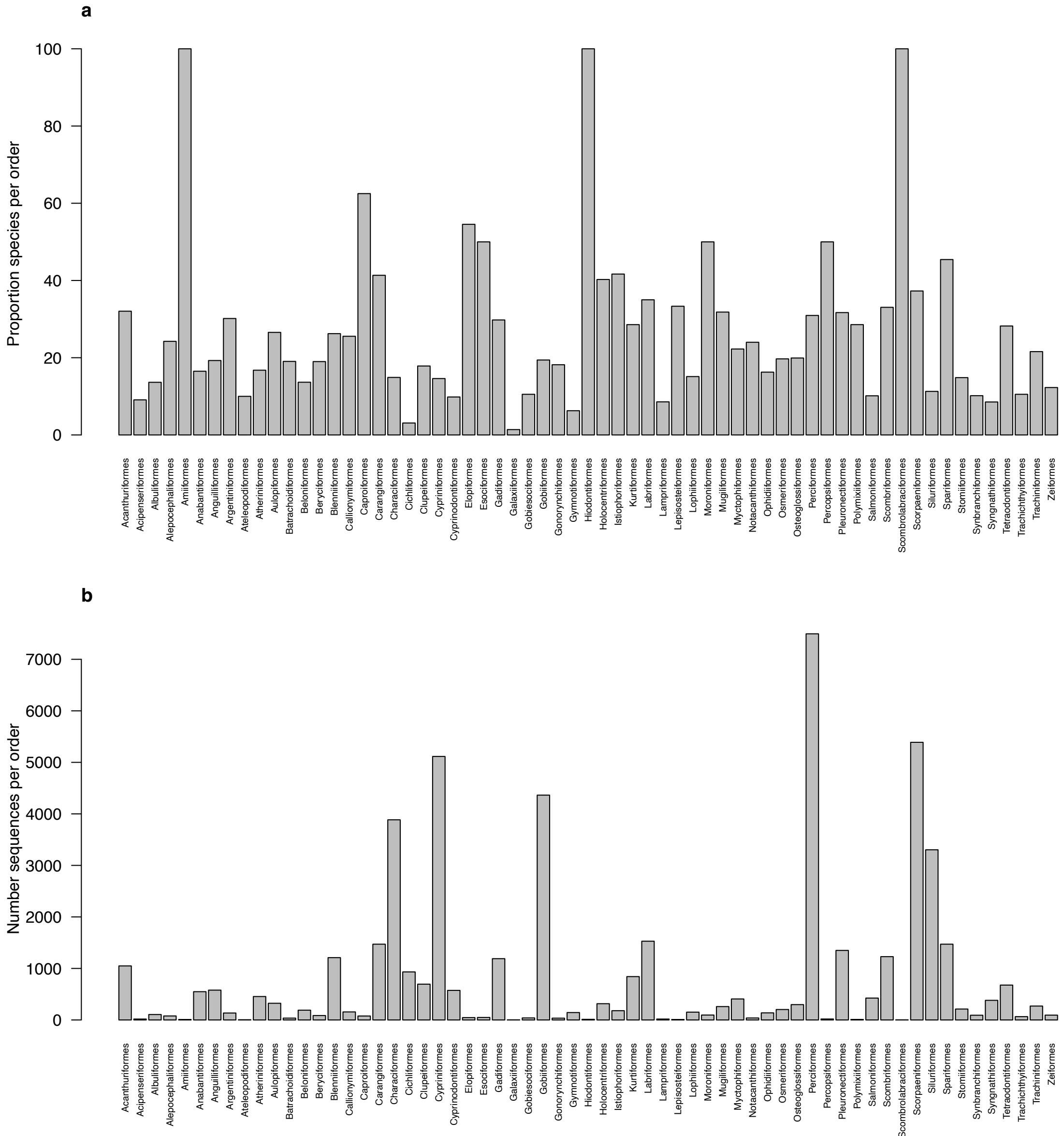

Supplementary Figure S5. Global taxonomic coverage of the 50,588 sequences used in the model (a) Number of species per order (b) Number of sequences per order

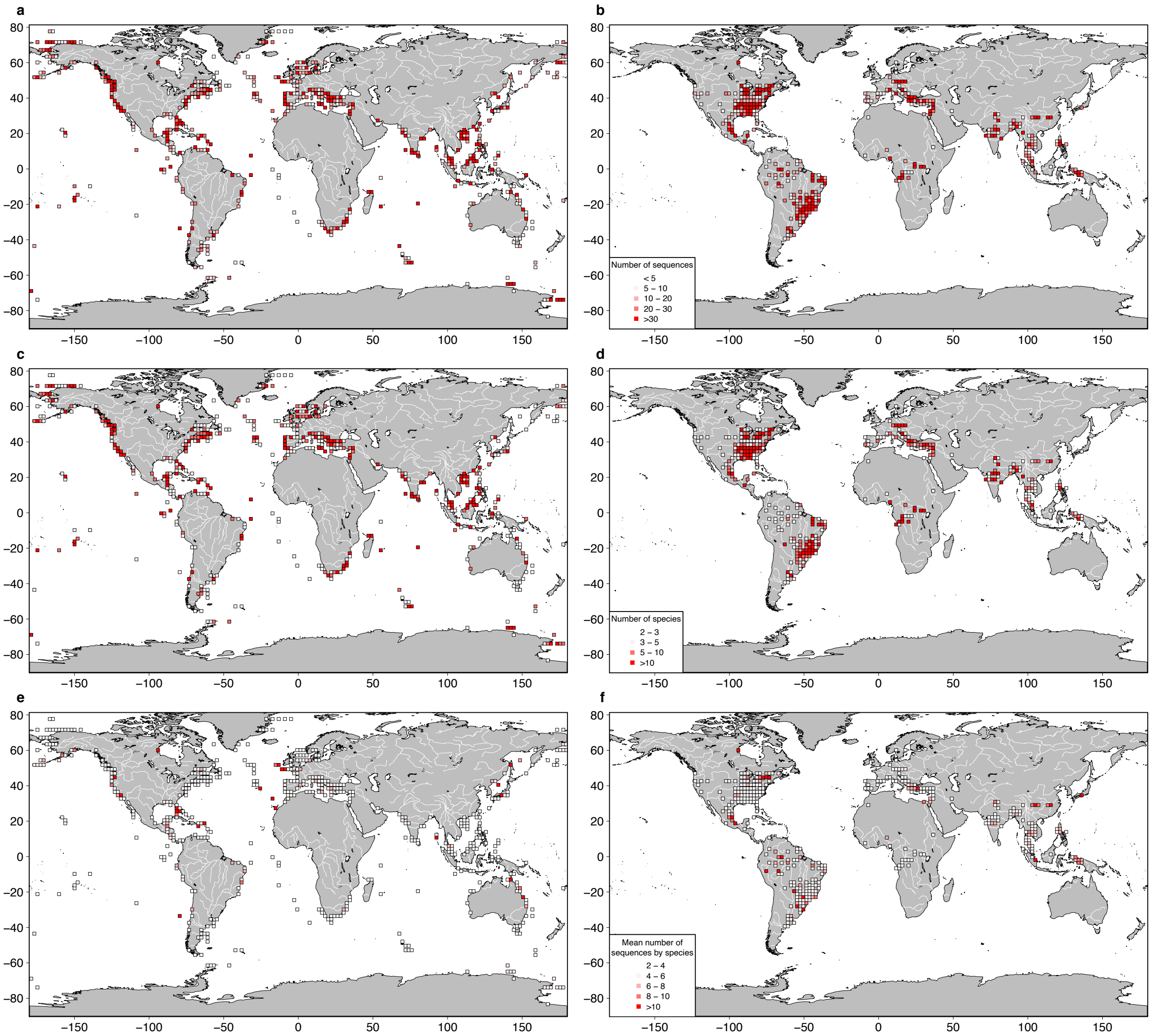

Supplementary Figure 6. Sampling effect. (a,b) number of sequences per cell. Colour scales is defined in (b). (c,d) Number of fish species per cell. Colour scales is defined in (d). (e,f) Mean number of sequences per fish species per cell for marine species. Colour scales is defined in (f). (a, c, e) Marine and (b, d, f) and freshwater species respectively. Drawn with R version 3.2.3.

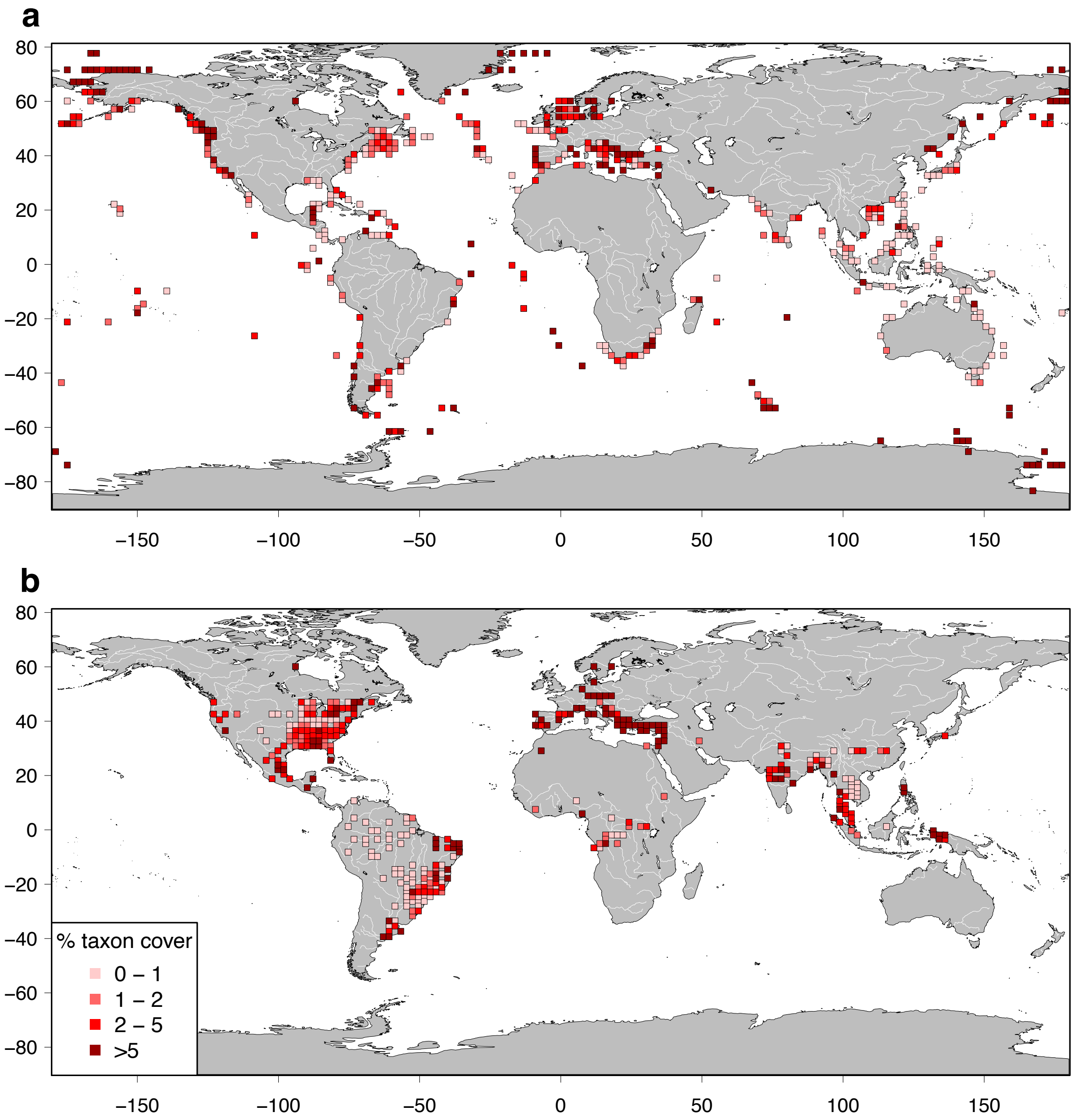

Figure S7. Taxonomic coverage estimated in each cell as the (number of species used to estimate genetic diversity)/species richness for (a) marine species (b) freshwater species. Colour scales is defined in (b). Drawn with R version 3.2.3.

Supplementary Tables

Supplementary Table 1. Description of the data used for our analysis. Raw genetic data are mitochondrial gene sequences from the Barcode of Life Database (BOLD; www.boldsystems.org; 09/17/2018). Number of sequences and species after each filtering steps.

	# Sequence	# species
**Raw data**	144867	11252
**Georeferenced (latitude/longitude)**	106196	8029
**known species**	72133	8029
**no IUAPC ambiguity**	64090	7702
**cytochrome oxidase subunit 1 5' region**	60260	7593
**>2 sequences by species**	58565	5912
**Model GD and factors**	50588	5426

Supplementary Table 2. Number cells, Mean number of sequences per species cell (Mean Seq) and of mean number of species per cell (Mean Spe) after each filtering steps

	Number of cells		Mean Seq		Mean Spe	
	**MR**	**FW**	**MR**	**FW**	**MR**	**FW**
**raw**	1028	577	4.4	4.3	7.6	5.5
**> 2 species per cell**	631	364	4.3	4.6	11.7	8.2
**Genetic diversity different from 0**	616	356	4.3	4.7	12	8.3
**SST/regional temperature available**	584	355	4.3	4.7	12.4	8.3
**CloMeanVal information available**	514	355	4.1	4.7	13.1	8.3
**Bathymetry information available**	514	355	4.1	4.7	13.1	8.3
**Correction for annotation error in FishBase**	514	346	4.1	4.7	13.1	8.5
**Species richness available**	455	340	4.1	4.7	13.4	8.5

MR: marine species
FW: Freshwater species

Supplementary Table 3.

Difference between marine and freshwater diversity both at intra and species level respectively. Results of the linear model between genetic and species diversity and two factors: latitude and habitat type- a binary variable indicating whether the sequence is marine (1) or freshwater (0). Regression coefficients and *p*-values of student tests are reported in the table. Diversity metrics were log-transformed and standardized to produce variables following a normal distribution before the linear regression. The relative variances of coefficients were estimated with the package hier.part and are reported in bracket (%).

	Genetic diversity		Species diversity	
	Coefficient	*p* - value	Coefficient	*p* - value
Species type	-0.40 (67)	0.07	0.04 (0.5)	0.51
Latitude	-0.004 (37)	0.001	-0.006 (99)	5.9×10^{-10}
Adjusted r^2	0.06		0.04	

Supplementary Table 4. Description of data sources

A value of bathymetry and minimal, maximum and average chlorophyll were assigned for each cells of the grid for marine species only.

Variable category	Names	Source and units
Geographic (*Z*)	Latitude, Longitude[1]	BOLD (degree)
	Bathymetry[2,3]	The GEBCO_2014 Grid, version 20150318, www.gebco.net
	Region	Atlantic vs Indo-pacific for marine species and Africa, Antarctica, Europe, North America, Oceania and South America for freshwater species
	Distance from offshore[2]	http://gmed.auckland.ac.nz/download.html (km)
	Basin area[4]	https://www.nature.com/articles/sdata2017141 (m^2)
	slope (avg, range) and flow accumulation[4]	http://www.earthenv.org/streams<br>[°] * 100 and Count of grid cells
Environmental (*Y*)	Temperature (SST)[5]	Marine: http://gmed.auckland.ac.nz (°C)<br>Freshwater species: http://www.worldclim.org/ (°C)
	Oxygen concentration	http://gmed.auckland.ac.nz (mg/l)
	Chlorophyll[2]	www.oracle.ugent.be (mg/m3)
Sampling (*S*)	Number of species per cells	https://gitlab.mbb.univ-montp2.fr/reservebenefit/worldmap_fish_genetic_diversity/
	Number of sequences per species per cells	https://gitlab.mbb.univ-montp2.fr/reservebenefit/worldmap_fish_genetic_diversity/

1-Latidude and Longitude were used to calculate the autocovariate variable.

2-Only for marine species. Bathymetry values were obtained by overlaying and averaging bathymetry data from GEBCO's gridded bathymetric data sets at a resolution of 30 arc-seconds (raster) with the grid layer (vector) using the extract function from raster package in R. We used the same methodology to extract average chlorophyll-a values (called respectively bathymetry and chlorophyll in our analysis) [1]. The information of chlorophyll-a was obtained from the Bio-ORACLE database at a resolution of 5 arc-minutes. Chlorophyll-a and Oxygen were removed for freshwater species because of too high number of missing values.

3- Bathymetry refers to elevation for freshwater species. For model analysis, we replaced negative values of altitudes by 0 for concerned freshwater species (e.g. coastal species, lacustrine…).

4-Only for freshwater species. Values were obtained from Domisch et al [2] and are available from a grid of resolution 1 km. For our analysis, we averaged all the 1 km pixels in each 200 km cell of our grid y using the extract function from the package raster. Flow accumulation is the amount of upstream area draining into each cell and is measured in count of grid cells. It could be considered as a surrogate of watershed size. Slope Units: ([°] * 100). Slopes values

(average and range in our analysis) are estimated from the upstream slope of each cell of the 1km grid. More details on the variables can be found in Domisch *et al.* [2].

5-For marine species: sea surface temperature; for freshwater species: regional mean temperature (1970-2000 resolution ~340 km^2)

Supplementary Table 5: Selection of factors in the models between the genetic diversity average across species in each cell, and geography (*Z*) and environment (*Y*). Sampling (*S*) factors were always kept in the models to account for data structure. The table provides the outputs of the AIC procedure using the R function stepAIC with the backward procedure, after previous selection of factors based on VIF > 5. We manually recalculated the AIC by keeping number of species and sequences when removed. In bold the final model selected by the AIC procedure. All environmental factors were standardized before analysis.
GD_mean: SST: sea surface temperature for marine species and regional air temperature for freshwater species; nb_species : number of species, nb_indv : number of individuals; Only for marine species: bathyVal: bathymetry, cloMeanVal: chorophyll ; distanceFromShore : distance from shore. Only for Freshwater species : Alt: elevation; SlopeAvg: average slope.Flow accumulation and basin area were tested on a reduced dataset because of too missing values (20%).

**Marine species**	AIC
GD_mean ~ SST + bathyVal + cloMeanVal + nb_species +nb_indv_mean + distanceFromShore+regions + autocor	-71.26
GD_mean ~ SST + cloMeanVal + nb_species + nb_indv_mean + regions + autocor	-73.26
**GD_mean ~ SST + nb_species + nb_indv_mean + regions + autocor**	**-74.97**
**Freshwater species**	
GD_mean ~ SST + regions + Alt +SlopeAvg+ nb_species + nb_indv_mean++ autocor	-74.46
**GD_mean ~ SST + regions + autocor+SlopeAvg+ nb_species + nb_indv_mean**	**-75.13**

Supplementary Table 6.

Summary of the linear models testing the effect of geographic, environmental, and sampling effect as on fish genetic diversity. We added a term to control for spatial autocorrelation (autocor) in the model. Only variables selected after VIF procedure and stepwise selection are reported (VIF>5). For marine species, the factors bathymetry, chlorophyll, oxygen were removed after VIF procedure and stepwise selection procedure and were not kept in the final model. For freshwater basin area, elevation, slope range and flow accumulation were not selected after VIF procedure and stepwise selection. Values reported in the table are regression coefficients, values in brackets are standard errors estimated from the lm, Asterisks indicate the level of significance (*** $p < 0.001$, ** $p < 0.01$, * $p < 0.05$), and p-values of the student test on the coefficient regression. For marine species, after filters for species, sequence numbers, and missing values, 514 cells were used in the model, and 343 for freshwater species. We visually checked for normality and independence of residuals of both models. We found no spatial autocorrelation in the residuals for both models (Moran I = -0.05, p = 0.76 for marine species and Moran I = -0.018, p = 0.573 for freshwater species).

	Marine species		Freshwater species	
	Coefficient	*p* -value	Coefficient	*p*- value
Temperature	0.34 (0.04) ***	2x84-16	0.13 (0.058) **	0.02
Slope avg	/	/	-0.24 (0.051)**	1.18x10-5
Region	0.22 (0.08) **	0.007	0.12 (0.028)***	2.31x10-5
Autocor	0.076 (0.04)	0.062	0.24 (0.048)***	1.26x10-6
Species number	0.003 (0.0013) *	0.034	0.004 (0.0050)	0.37
Sequence number	-0.083 (0.04)	0.055	0.020 (0.0486)	0.68
Adjusted r^2	0.16	2x10-16	0.19	7.62x10-16

Supplementary Table 7. Sensitivity analysis for the correlation between genetic diversity and species diversity. We investigated the impact of the amount of genetic data and of taxonomic coverage on the correlation between genetic diversity and species richness both on marine and freshwater species. In practice, we first, increased the number of sequences or the number of species in each cell of the grid and keep only grid cells with top values (about top 1/3 or 2/3 cells). Then for fixed number of sequences and species, we varied the taxonomic coverage estimated in each cell as (the number of species used for genetic diversity estimation/cell species richness). Values reported in the table are the modified t- test of the correlation between genetic and species diversity and associated p-values. Significant values are indicated in red. The data considered as reference is the one with 514 cells (Supplementary Table 2).

For marine species, after filters for species, sequence numbers, and missing values, 455 cells were used in the analysis, and 340 for freshwater species

	**Marine species**							
**Sequence (≥)**	2	2	3	4	2	2	2	2
**Species (≥)**	3	8	2	2	2	2	2	2
**Taxonomy (%)**	0	0	0	0	0	1	2	5
**Cells (number)**	340	166	293	153	455	322	232	146
**Cells (%)**	74	37	64	33	100	71	51	32
**Modified t-test**	0.26	0.51	0.20	0.30	0.21	0.12	0.10	0.06
***p*-value**	0.05	0.02	0.08	0.05	0.01	0.05	0.19	0.41
	**Freshwater species**							
**Sequence**	2	2	3	5	2	2	2	2
**Species**	3	8	2	2	2	2	2	2
**Taxonomy(%)**	0	0	0	0	0	1	2	5
**Cells (number)**	257	117	256	111	340	233	185	100
**Cells (%)**	75	34	75	32	100	68	54	29
**Modified t-test**	0.41	0.42	0.43	0.37	0.36	0.11	0.12	0.18
***p*-value**	0.001	0.01	0.01	0.12	0.01	0.30	0.27	0.13

Supplementary Table 8. Sensitivity analysis of the model (linear relation between genetic diversity and significant factors). We investigated the impact of the amount of genetic data and of taxonomic coverage per cell on model outputs. In practice, we first, increased the number of sequences or the number of species in each cell of the grid and keep only grid cells with top values (about top 1/3 or 2/3 cells). Then for fixed number of sequence and species, we varied taxonomic coverage estimated in each cell as (number of species for genetic diversity estimation/cell species richness). Values in the table are the regression coefficients for those new models. CV (%) is the coefficient variation between the new models and the model reported in Table S5 in all case. Taxonomic coverage per cell can only be evaluated for 455 cells and 340 cells respectively for marine and freshwater species because of missing values for species richness in the other cells. Positives values indicate an increase of the new coefficient and negative values a decrease.

	**Marine species**							
	Filter							
**Sequence (>)**	2	2	3	4	2	2	2	2
**Species (≥)**	3	8	2	2	2	2	2	2
**Taxonomy (%)**	0	0	0	0	0	1	2	5
**Cells (number)**	383	186	339	179	455[1]	322	232	146
**Cells (%)**	74	36	66	34	88	62	45	28
	Model							
Temp	0.38	0.38	0.33	0.32	0.33	0.35	0.32	0.25
CV Temp	12	10	-4	-6	-1	3	-7	-27
Region	0.21	0.32	0.22	0.25	0.17	0.28	0.18	0.11
CV Region	-3	44	-2	14	21	30	-16	-48
Autocor	0.09	0.01	0.09	0.02	0.14	0.14	0.12	0.05
CV Autocor	17	-77	25	-79	-87	87	53	-32
Adjusted r^2	0.23	0.34	0.17	0.12	0.17	0.20	0.17	0.11
CV Adjusted r^2	43	100	8	-21	6	25	7	-27
	**Freshwater species**							
	Filter							
**Sequence (>)**	2	2	5	3	2	2	2	2
**Species (≥)**	3	8	2	2	2	2	2	2
**Taxonomy (%)**	0	0	0	0	0	1	2	5
**Cells (number)**	262	118	113	260	340	233	185	100
**Cells (%)**	75	34	32	75	98	67	53	29
	Model							
**Temp**	0.10	0.12	0.03	0.14	0.15	0.08	0.04	-0.01
**CV temp**	-26	-8	-79	7	13	-37	-63	-100
**Slope avg**	-0.20	-0.16	-0.06	-0.16	-0.22	-0.19	-0.17	-0.11
**CV Slope avg**	-11	-27	-69	-27	-1	-12	-21	-50

**Region**	0.14	0.09	0.26	0.17	0.12	0.09	0.13	0.17
**CV Region**	23	-24	110	40	-2	-20	9	42
**Autocor**	0.21	0.11	0.30	0.20	0.23	0.25	0.23	0.21
**CV Autocor**	-7	-54	25	-13	-4	3	0	-9
**Adjusted r^2**	0.24	0.22	0.29	0.22	0.19	0.20	0.21	0.21
**CV Adjusted r^2**	26	14	49	12	0	3	6	9

1-The number of cells was reduced from 514 to 455 because of the filter on taxonomic coverage.

Supplementary Table 9 – Global taxonomy coverage
Quartile of the proportion of species and number of sequences for the 63 orders and 480 families described for fish in NCBI. The quartiles are given for the 57703 sequences and 5426 species used for the map (Fig. 1) and in the model (Fig. 3). Those number are provided globally across all cells of the grid.

	0	**Min**	**25%**	**50%**	**75**	**Max**
**Species proportion (%)**						
Order		1.39[1]	14.13	21.58	32.55	100[2]
Family		0	0	18.25	36.28	100[4]
**Sequence Number**						
Order		2	57	203	768	7495[5]
Family		0	0	14	73	4515[6]

1-The number of species is the lowest for the Galaxiiformes order.
2- *Amiiformes*, *Hiodontiformes* and *Scombrolabraciformes* are fully represented orders with 100 % of species present in the dataset.
3-135 families have no species
4- 24 families are fully represented.
5-The Perciformes is the order with the highest number of sequences.
6-The Cyprinidae is the family with the highest number of sequences

Supplementary References

1. Tyberghein L, Verbruggen H, Pauly K, Troupin C, Mineur F, De Clerck O. Bio-ORACLE: a global environmental dataset for marine species distribution modelling. *Global Ecology and Biogeography* **21**, 272-281 (2012).

2. Domisch S, Amatulli G, Jetz W. Near-global freshwater-specific environmental variables for biodiversity analyses in 1 km resolution. *Scientific Data* **2**, 150073 (2015).
