# Supplementary material for: Global determinants of freshwater and marine fish genetic diversity
Source: Nat Commun. 2020 Feb 10;11:692. doi: 10.1038/s41467-020-14409-7 (PMC7010757; doi:10.1038/s41467-020-14409-7)
Supplement: Supplementary file 3 — Reporting Summary [file 41467_2020_14409_MOESM3_ESM.pdf]

nature research

Corresponding author(s): Stéphanie Manel

Last updated by author(s): 24/12/2019

Reporting Summary

Nature Research wishes to improve the reproducibility of the work that we publish. This form provides structure for consistency and transparency in reporting. For further information on Nature Research policies, see Authors & Referees and the Editorial Policy Checklist .

Statistics

For all statistical analyses, confirm that the following items are present in the figure legend, table legend, main text, or Methods section.

n/a	Confirmed	
☐	☒	The exact sample size (*n*) for each experimental group/condition, given as a discrete number and unit of measurement
☒	☐	A statement on whether measurements were taken from distinct samples or whether the same sample was measured repeatedly
☐	☒	The statistical test(s) used AND whether they are one- or two-sided<br>*Only common tests should be described solely by name; describe more complex techniques in the Methods section.*
☐	☒	A description of all covariates tested
☐	☒	A description of any assumptions or corrections, such as tests of normality and adjustment for multiple comparisons
☐	☒	A full description of the statistical parameters including central tendency (e.g. means) or other basic estimates (e.g. regression coefficient) AND variation (e.g. standard deviation) or associated estimates of uncertainty (e.g. confidence intervals)
☐	☒	For null hypothesis testing, the test statistic (e.g. *F*, *t*, *r*) with confidence intervals, effect sizes, degrees of freedom and *P* value noted<br>*Give P values as exact values whenever suitable.*
☒	☐	For Bayesian analysis, information on the choice of priors and Markov chain Monte Carlo settings
☒	☐	For hierarchical and complex designs, identification of the appropriate level for tests and full reporting of outcomes
☒	☐	Estimates of effect sizes (e.g. Cohen's *d*, Pearson's *r*), indicating how they were calculated

Our web collection on statistics for biologists contains articles on many of the points above.

Software and code

Policy information about availability of computer code

Data collection	No software was used to collect the data.
Data analysis	All statistical analyses have been conducted with the version 3.5,1 of the R software. All the scripts are available in the worldmap_fish_genetic_diversity repository:<br>https://gitlab.mbb.univ-montp2.fr/reservebenefit/worldmap_fish_genetic_diversity

For manuscripts utilizing custom algorithms or software that are central to the research but not yet described in published literature, software must be made available to editors/reviewers. We strongly encourage code deposition in a community repository (e.g. GitHub). See the Nature Research guidelines for submitting code & software for further information.

Data

Policy information about availability of data

All manuscripts must include a data availability statement. This statement should provide the following information, where applicable:

- Accession codes, unique identifiers, or web links for publicly available datasets
- A list of figures that have associated raw data
- A description of any restrictions on data availability

The datasets generated during and/or analysed during the current study are available in the worldmap_fish_genetic_diversity repository, https://gitlab.mbb.univ-montp2.fr/reservebenefit/worldmap_fish_genetic_diversity. The source data underlying Figs. 1b, d, 2a, c, 3a, c and Supplementary Figs 1 to 7 are provided as a Source Data file.

Field-specific reporting

Please select the one below that is the best fit for your research. If you are not sure, read the appropriate sections before making your selection.

☐ Life sciences ☐ Behavioural & social sciences ☒ Ecological, evolutionary & environmental sciences

For a reference copy of the document with all sections, see nature.com/documents/nr-reporting-summary-flat.pdf

Ecological, evolutionary & environmental sciences study design

All studies must disclose on these points even when the disclosure is negative.

Study description	This study investigated a linear model to explore the relationship between fish genetic diversity and three types of quantitative factors among which Y are environmental, Z geographic and S sampling variables. We also included a spatial autocovariate to take into account the spatial structure in our data. Fish genetic diversity was estimated in each cell of a grid covering the study area using the sample described below (i.e. individual sequence).
Research sample	Raw genetic data (research sample) are fish mitochondrial gene sequences from the Barcode of Life Database (BOLD; www.boldsystems.org).
Sampling strategy	Sample size was the number of sequences used to estimate genetic diversity in our analysis. It was obtained after different steps of filtering described in data removing section. Sample size is: 58565 sequences (distributed among 5912 species).
Data collection	PE Guerin collected the data from Barcode of Life Database (BOLD; www.boldsystems.org)
Timing and spatial scale	Data were collected in one time (09/17/2018) at www.boldsystems.org at
Data exclusions	Different filters were applied to the raw collected data: only sequence longer than 500bp and annotated as Cytochrome Oxidase Subunit 1 - 5' Region (CO1) from the Barcode of Life Database with species, coordinates (latitude, longitude) and region name information were included in the final sample. Species with only one sequence (i.e. one individual) were also removed.
Reproducibility	All statistical analyses were reproduced 3 times.
Randomization	This is not relevant for our study. Our study is an observation study in the field of biogeography. We used a random factor (autocorrelated variable) to control for spatial autocorrelation.
Blinding	Blinding is not relevant in our case. We used only known sequences and there is no experiment.

Did the study involve field work? ☐ Yes ☒ No

Reporting for specific materials, systems and methods

We require information from authors about some types of materials, experimental systems and methods used in many studies. Here, indicate whether each material, system or method listed is relevant to your study. If you are not sure if a list item applies to your research, read the appropriate section before selecting a response.

Materials & experimental systems

n/a	Involved in the study	
☒	☐	Antibodies
☒	☐	Eukaryotic cell lines
☒	☐	Palaeontology
☒	☐	Animals and other organisms
☒	☐	Human research participants
☒	☐	Clinical data

Methods

n/a	Involved in the study	
☒	☐	ChIP-seq
☒	☐	Flow cytometry
☒	☐	MRI-based neuroimaging
